# Ezrin expression combined with MSI status in prognostication of stage II colorectal cancer

**DOI:** 10.1371/journal.pone.0185436

**Published:** 2017-09-27

**Authors:** Khadija Slik, Samu Kurki, Taina Korpela, Olli Carpén, Eija Korkeila, Jari Sundström

**Affiliations:** 1 Department of Pathology, University of Turku, Kiinamyllynkatu 10, Turku, Finland; 2 Department of Pathology, Misurata Cancer Center, University of Misurata, Faculty of Dentistry, Department of Basic Sciences, Misurata, Libya; 3 Auria Biobank, University of Turku and Turku University Hospital, Kiinamyllynkatu 8, Turku, Finland; 4 Department of Pathology and Genome Scale Biology Research Program, University of Helsinki and HUSLAB, Helsinki University Hospital, Helsinki, Finland; 5 Department of Oncology, Turku University Hospital, Hämeentie 11, Turku, Finland; 6 Department of Pathology, Turku University Hospital, Kiinamyllynkatu 10, Turku, Finland; National Cancer Center, JAPAN

## Abstract

Currently used factors predicting disease recurrence in stage II colorectal cancer patients are not optimal for risk stratification. Thus, new biomarkers are needed. In this study the applicability of *ezrin* protein expression together with *MSI* status and *BRAF* mutation status were tested in predicting disease outcome in stage II colorectal cancer. The study population consisted of 173 stage II colorectal cancer patients. Paraffin-embedded cancer tissue material from surgical specimens was used to construct tissue microarrays (TMAs) with next-generation technique. The TMA-slides were subjected to following immunohistochemical stainings: MLH1, MSH2, MSH6, PMS2, ezrin and anti-BRAF V600E antibody. The staining results were correlated with clinicopathological variables and survival. In categorical analysis, high ezrin protein expression correlated with poor disease-specific survival (p = 0.038). In univariate analysis patients having microsatellite instabile / low ezrin expression tumors had a significantly longer disease-specific survival than patients having microsatellite stable / high ezrin expression tumors (p = 0.007). In multivariate survival analysis, the presence of *BRAF* mutation was associated to poor overall survival (p = 0.028, HR 3.29, 95% CI1.14–9.54). High ezrin protein expression in patients with microsatellite stable tumors was linked to poor disease-specific survival (p = 0.01, HR 5.68, 95% CI 1.53–21.12). Ezrin protein expression is a promising biomarker in estimating the outcome of stage II colorectal cancer patients. When combined with microsatellite status its ability in predicting disease outcome is further improved.

## Introduction

Five-year survival in stage II colorectal cancer (CRC) is 70–80% [1,2]. Unfavorable prognostic factors for stage II CRC include lymphovascular invasion, less than 12 examined lymph nodes, poor differentiation grade, tumor spreading to the peritoneum or adjacent tissue structures as well as tumor obstruction or perforation. [3,4]. These risk factors have been utilized in the assessment of stage II colorectal cancer patients in need of postoperative adjuvant treatments. The benefit of chemotherapy in stage III colorectal cancer patients is apparent, but controversial in stage II colorectal cancer patients even with above-mentioned risk factors [5]. Consequently, there is a crucial need to discover new markers to better define those at highest danger of disease recurrence.

DNA mismatch repair competence is a feature associated with CRC outcome. Inactivation of genes responsible for mismatch repair competence cause microsatellite instability (*MSI*), which can be studied by expression of the gene products MLH1, MSH2, MSH6 and PMS2 or by PCR-based methods [6,7,8,9,10,11]. *MSI* is reported in about 15–20% of CRC [8,12]. *MSI* is linked to right-sided, poorly differentiated tumors with higher T stage and younger patient age [12]. Stage II CRCs with *MSI*, as demonstrated by immunohistochemistry of mismatch repair proteins, have a more favorable prognosis as compared to microsatellite stable (*MSS*) tumors [13,14,4]. Moreover, patients with defective mismatch repair (dMMR) stage II tumors do not seem to benefit from fluorouracil-based adjuvant chemotherapy [15,16]. *MSI* tumor can evolve in Lynch syndrome patients carrying a germ-line mutation in one MMR gene, or through sporadic events involving epigenetic silencing of the *MLH1* gene [17].

*BRAF* gene encodes a protein kinase of the *RAS/RAF/MEK-ERK* signaling cascade, which is regulated by *KRAS* [18,19]. Previously, *BRAF* V600E mutation was shown to be an adverse prognostic factor for overall survival in stage II-III colon cancer [20]. *MSS* together with *BRAF* mutation is associated with poor prognosis in CRC [9,21]. On the contrary, *MSI* stage II tumors with *BRAF V600E* mutation are associated with a rather favorable prognosis [21]. Moreover, colorectal cancer with *MSI* phenotype and a concomitant *BRAF* mutation indicates a sporadic tumor, thus excluding Lynch syndrome [17,21].

Ezrin is a cytoskeleton-associated protein, which participates in cellular signaling, cell survival, proliferation and migration. Its association with malignant behavior has been suggested in several experimental models, and in several cancers strong ezrin expression correlates with inferior outcome. [22,23,24,25,26,27,28]. Our previous work has demonstrated the impact of ezrin expression on the outcome in metastatic CRC as well as in localized rectal cancer [27,28]. To our knowledge, the role of ezrin as a prognostic marker in stage II colorectal cancer has not been studied before.

In this work, we utilized tumor tissue collection form consecutive stage II CRC patients, together with extensive clinical, disease outcome and follow-up data to search for tissue-based prognostic markers. We report the association of *MSI* status, *BRAF* mutation status and ezrin protein expression with clinicopathological variables and patient outcome. Our results suggest that combined *MSI* and ezrin analysis can stratify tumors according to their clinical behavior.

## Patients and methods

### Study population

We collected archived paraffin-embedded tumor material from consecutive stage II CRC patients operated in Turku University Hospital in 2005–2012. This study was approved by Chief Executive Officer of TYKS-SAPA, Hospital District of Southwest Finland (T52/2014). The use of tissue material was approved by Scientific Steering Group of Auria Biobank (AB15-8108, 25.5.2012). The study was conducted in accordance with the Declaration of Helsinki. The clinical data were retrieved and histological samples collected and analyzed with the endorsement of the National Authority for Medico-Legal Affairs (VALVIRA). The patient records were accessed anonymously.

In 2005–2012 a total of 232 stage II CRC patients were radically operated in our hospital. Computed tomography (CT) of the abdomen and chest x-ray or CT had been performed preoperatively to rule out distant metastases. We carefully checked the patient files, including surgery and pathology reports and excluded patients with verified lymph node or distant metastases, those who had been operated with palliative-intent surgery, and also patients with other than adenocarcinoma histology (e.g. neuroendocrine tumors). Only patients with stage II CRC were included in the current study. For tumor staging, TNM7 classification of malignant tumors [29] was used. From the original cohort (n = 232), tumor material for *MSI* staining was available from 214 patients. For further BRAF and ezrin stainings, material was available from 173 patients. These patients (n = 173) were included in statistical analyses.

### TMA construction

Tissue microarrays (TMA) were constructed and analyzed using the next-generation TMA technique [30]. Shortly, the appropriate formalin-fixed paraffin-embedded (FFPE) tissue specimens were chosen based on clinical data and retrieved from the pathology archives. A representative hematoxylin-eosin (H&E) section containing areas of invasive carcinoma was selected from each tumor. New H&E slides were produced, scanned (Pannoramic P250, 3DHistech) and uploaded into the university digital microscopy web portal (casecenter.utu.fi). Each slide was viewed using Pannoramic Viewer software (3DHistech). Using the 1.2 mm diameter annotation tool, annotations of different colors corresponding to various histological areas were placed onto each digital slide. Two annotations were placed in the center of the tumor, two in the tumor front and two in the normal colonic epithelium. The corresponding tissue cores were then transferred into the TMA blocks using an automated TMA instrument (TMA Grandmaster, 3DHistech) by overlaying each annotated digital slide with the corresponding tissue specimen. One tissue core containing benign tissue was selected from each tumor to act as a control. The constructed TMA blocks were sectioned, stained, scanned and uploaded into the web portal (casecenter.utu.fi) and each individual spot was scored by two pathologists (KS, JS). The resulting scores were combined with the clinical data for statistical analysis.

### Immunohistochemistry

Immunohistochemical staining against MMR proteins is a useful screening method in research materials with paraffin-embedded TMA-samples. In contrast to PCR-based methods, it also readily provides information on the inactivated gene. Immunohistochemical stainings (IHC) were performed using standard procedures. Shortly, 3,5 μm sections were cut from the TMA blocks. They were stained with monoclonal antibodies against MLH1 (Clone G168-15BD Pharmingen, dilution: 1:5), MSH2 (Clone G219-1129, BD Pharmingen, dilution: 1:200) and MSH6 (Clone EP49, Epitomoc, dilution: 1:200). The signal was detected with UltraView Universal DAB Detection kit. For PMS2, Clone EPR3947 (Ventana/Roche, ready to use antibody) was used and the signal was detected with OptiView Universal DAB Detection Kit and amplification kit. To detect *BRAF V600E* mutation, BRAF RTU antibody (Clone VE1, Roche/Ventana) was used and the signal was detected with OptiView Universal DAB Detection kit. For ezrin staining, immunoglobulin G antibody to human ezrin (clone 3C12) [31] was used. All the stainings were performed with BenchMark XT (Ventana/Roche) using ultraVIEW Universal DAB Detection Kit (Ventana/Roche), except ezrin, which was done with LabVision immunoautomate (Thermo Fisher Scientific) using the Power Vision Plus poly HRP anti-mouse/rabbit/rat IgG detection kit.

### Evaluation of immunohistochemical stainings

All IHC stainings were separately evaluated by two observers (KS and JS), blinded to clinical data. For MLH1, MSH2, MSH6, PMS2 and ezrin, inflammatory cells of the stroma were used as positive controls. For analyses of *MSI* (MLH1, MSH2, MSH6 and PMS2) also the cores from normal colonic epithelium were used as positive controls. As a positive control in evaluating the BRAF-stainings, we used *BRAF V600E* mutation-positive cancer tissue obtained from a CRC patient who did not belong to this study cohort. These IHC stainings were evaluated dichotomously as positive or negative. For ezrin protein expression, cytoplasmic staining was recorded [27, 28].Four staining categories were used: 0 for negative staining, 1 for weak staining (distinguishable from the background staining), 2 for moderate staining and 3 for strong staining (corresponding to immunoreactivity in lymphocytes) [27]. In addition, a category of non-evaluable was used for all stainings. For statistical purposes a dichotomous grading, ezrin low (negative or weak staining) and ezrin high (moderate or strong staining) was used.

### Statistical analysis

Statistical analyses were performed with IBM SPSS version 23 with standard packages. Clinical data were analyzed in correlation with histological, immunohistochemical and mutational analysis data using χ^2^ or Fisher’s exact-test for discrete variables and one-way ANOVA for continuous variables. Overall survival (OS), disease free survival (DFS) and disease-specific survival (DSS) were calculated using Kaplan-Meier curves. Survival was analyzed with respect to (stratified to) different biomarkers using log-rank test. For multivariate analyses, the following variables were used: tumor grade, tumor-side, obstruction, perforation, vascular invasion, *BRAF* mutated/wild type, ezrin low/high and *MSS/MSI* combinations were included. Multivariate Cox proportional hazard regression model was used to adjust the survival curves for covariates and to obtain estimates on hazard ratios. All p-values were two-sided, and values less than 0.05 were considered statistically significant.

## Results

### General aspects of clinical patient characteristics

Altogether 173 patients were included in this study. The tumor was located in the proximal colon in 70 (40%), transverse colon in 19 (11%), descending colon in 8 (5%), sigmoid colon in 44 (25%) and rectum/rectosigmoideum in 32 (19%) patients. There were 30 (17%) T4-tumors included in the study. Vascular invasion was reported in 32 (18%) patients and preoperative bowel obstruction in 26 (15%) of patients. Adjuvant fluorouracil-based chemotherapy had been given to 51 (30%) patients. The median follow-up time was 57 months. At the latest follow-up data collection time point in September 2016, 116 patients (67%) were alive without CRC, 3 (2%) alive with CRC, 17 dead of CRC, 18 (10%) dead of other cancers and 19 (11%) dead of other causes than cancer. The clinical characteristics of the patients are shown in Table 1.

**Table 1:** The clinicopathological variables of the patient population included in the MSI, BRAF and Ezrin analyses (n = 173). NA = not available, R0 = microscopically radical surgery, R1 macroscopically radical surgery, R2 macroscopically non-radical surgery.

Variable	n (%)
**Gender**	
Female	92 (53)
Male	81 (47)
**Age**	
<70 years	66 (38)
>70 years	107 (62)
**Postoperative stage**	
T3N0	143 (83)
T4aN0	17 (10)
T4bN0	13 (7)
**Tumor side**	
Right	89(51)
Left	84 (48)
**Tumor grade** (analyzed from surgical specimens)	
G1	19 (11)
G2	114 (66))
G3	40 (23)
**Histology**	
Conventional adenocarcinoma	151 (87)
Mucinous adenocarcinoma	22 (13)
**Vascular invasion**	
Yes	32 (18)
No	131 (76)
NA	10 (6)
**Lymph node count**	
≥12 lymph nodes examined	138 (80)
<12 lymph nodes examined	35 (20)
**Radicality**	
R0	162 (94)
R1	8 (5)
R2	3 (2)
**Preoperative obstruction**	
Yes	26 (15)
No	147 (85)
**Tumor perforation**	
Yes	15 (9)
No	157 (91)
NA	1 (0)
**Adjuvant chemotherapy**	
Yes	51 (30)
No	121 (69)
NA	1(0)

### General aspects of *MSI* staining

The results of the *MSS/MSI* analysis in relation to clinicopathological variables are shown in Table 2. Overall, 136 (79%) of the tumors were *MSS* and 37 (21%) were *MSI* high. *MSI* was significantly more common in the right-sided tumors (n = 30; 34%), as compared with the left-sided tumors (n = 7; 8%) (Pearson’s chi-square test, p = 0.0001). *MSI* was infrequent in well-differentiated tumors (1/39, 3%), but common in tumors with poor differentiation grade (15/39, 40%). Ten out of 22 (45%) mucinous cancers presented *MSI*. *MSI* status in relation to clinic-pathological variables is presented in Table 2.

**Table 2:** *MSS/MSI* status in relation to clinicopathological variables (n = 173). NA = not available, R0 = microscopically radical surgery, R1 = macroscopically radical surgery, R2 = macroscopically non-radical surgery, CRC = colorectal cancer.

Variable	*MSS*	*MSI high*	Significance *(p)*
	n (%)	n (%)	
**Gender**			0.266
Female	69 (51)	23 (62)	
Male	67 (49)	14 (38)	
**Age**		** **	0.707
Under 70 years	53 (39)	13 (35)	
Over 70 years	83 (61)	24 (65)	
**Postoperative stage**			0.253
T3N0	115 (85)	28 (76)	
T4aN0	13 (10)	4 (11)	
T4bN0	8 (6)	5 (13)	
**Tumor side**			**0.0001**
Right	59 (43)	30 (81)	
Left	77 (57)	7 (19)	
**Tumor grade**			**0.010**
G1	18 (13)	1 (3)	
G2	93 (68)	21 (57)	
G3	25 (18)	15 (40)	
**Histology**			**0.009**
Conventional adenocarcinoma	124 (91)	27 (73)	
Mucinous adenocarcinoma	12 (9)	10 (27)	
**Vascular invasion**			0.383
Yes	28 (21)	4 (11)	
No	101 (74)	30 (81)	
NA	7 (5)	3 (8)	
**Lymph node count**			0.646
12 or more examined	107 (78)	31 (84)	
Less than 12 examined	29 (21)	6 (16)	
**Radicality of surgery**			0.446
R0	128 (94)	34 (92)	
R1	5 (4)	3 (8)	
R2	3 (2)	0 (0)	
**Preoperative obstruction**			0.604
Yes	22 (16)	4 (11)	
No	114 (84)	33 (89)	
**Tumor perforation**			0.797
Yes	11 (8)	4 (11)	
No	124 (91)	33 (89)	
NA*	1 (1)	0 (0)	
**Adjuvant chemotherapy**			0.218
Yes	37 (27)	14 (38)	
No	99 (73)	22 (59)	
NA*	0 (0)	1 (3)	
**Disease-specific outcome**			0.660
Alive without CRC	93 (68)	23 (62)	
Alive with CRC	3 (2)	0 (0)	
Dead of CRC	13 (10)	4 (13)	
Dead of other cancer	13 (10)	5 (13)	
Dead of other causes	10 (7)	5 (13)	
Dead cause unspecified	4 (3)	0 (0)	

### General aspects of *ezrin* staining

The results of the ezrin stainings in relation to clinicopathological parameters are shown in Table 3. Generally, in 135 (78%) tumors, ezrin staining intensity was scored as low, and in 38 (28%) as high. High ezrin expression was more common in *MSI* tumors (19/37, 51%) than in *MSS* tumors (19/134, 14%) (Pearson’s chi-square test, p = 0.0001). There were no statistically significant differences in ezrin intensity according to clinicopathological variables, except for disease outcome (see below). Ezrin staining in relation to clinic-pathological variables is presented in Table 3.

**Table 3:** Ezrin expression in relation to clinicopathological variables (n = 173). CRC = colorectal cancer.

Variable	Ezrin low	Ezrin high	Significance *(p)*
	n (%)	n (%)	
**Gender**			0.272
Female	60 (44)	21 (55)	
Male	75 (56)	17 (45)	
**Age**		** **	0.130
Under 70 years	56 (41)	10 (26)	
Over 70 years	79 (58)	28 (74)	
**Postoperative stage**			0.634
T3N0	113 (84)	30 (79)	
T4aN0	13 (10)	4 10)	
T4bN0	9 (7)	4 (10)	
**Tumor side**			0.141
Right	65 (48)	24 (63)	
Left	70 (52)	14 (37)	
**Tumor grade**			0.119
G1	14 (10)	5 (13)	
G2	94 (70)	20 (53)	
G3	27 (20)	13 (34)	
**Histology**			0.099
Conventional adenocarcinoma	121 (90)	30 (79)	
Mucinous adenocarcinoma	14 (10)	8 (21)	
**Vascular invasion**			0.677
Yes	26 (19)	6 (16)	
No	100 (74)	31 (82)	
NA	9 (7)	1 (3)	
**Lymph node count**			1.000
12 or more examined	108 (80)	30 (79)	
Less than 12 examined	27 (20)	8 (21)	
**Radicality of surgery**			0.568
R0	127 (94)	35 (92)	
R1	6 (4)	2 (5)	
R2	1 (1)	1 (3)	
**Preoperative obstruction**			0.607
Yes	19 (14)	7 (18)	
No	116 (86)	31 (82)	
**Tumor perforation**			0.476
Yes	10 (7)	5 (13)	
No	124 (92)	33 (87)	
NA*	1 (1)	0 (0)	
**Adjuvant chemotherapy**			1.000
Yes	40 (30)	11 (29)	
No	95 (70)	26 (68)	
NA*	0 (0)	1 (3)	
***MSI status***			**0.001**
*MSS*	117 (87)	19 (50)	
*MSI*	18 (13)	19 (50)	
***BRAF status***			**0.001**
*BRAF WT*	121 (91)	25 (66)	
*BRAF mutated*	12 (9)	13 (34)	
**Disease-specific outcome**			**0.038**
Alive without CRC	93 (69)	23 (61)	
Alive with CRC	3 (2)	0 (0)	
Dead of CRC	8 (6)	9 (24)	
Dead of other cancer	16 (12)	2 (5)	
Dead of other causes	11 (8)	4 (11)	
Dead cause unspecified	4 (3)	0 (0)	

### BRAF staining

BRAF staining was available from 171 patients. Of the tumors, 146 (85%) were *BRAF wild type* and 25 (15%) *BRAF V600E mutated*. The *BRAF mutated* tumors predominantly presented with *MSI* (21/25, 84%), whereas *BRAF wild type* tumors were mostly *MSS* (130/146, 89%). Of the *BRAF wild type* tumors only 25/146 (17%) showed high ezrin IHC, while 13/25 (52%) of *BRAF mutant* tumors were ezrin high (Pearson’s chi-square test, p = 0.0001). Combinatorial analysis of the three variables showed that *BRAF wild type* tumors were predominantly *MSS* / low ezrin (112/146, 77%), whereas 12/25 (48%) of the *BRAF mutated* tumors were *MSI /* high ezrin (Fisher’s exact test, p = 0.0001). Follow-up data according to BRAF status is presented in Table 4.

**Table 4:** BRAF status in relation to ezrin and MSS/MSI (n = 171).

Variable	Ezrin MSS/Ezrin/MSI				Significance
					(p)
	***Ezrin low MSS***	***Ezrin low MSI***	***Ezrin high MSS***	***Ezrin high MSI***	**p = 0.0001**
	**n (%)**	**n (%)**	**n (%)**	**n (%)**	
***BRAF mutated***	3 (3)	9 (50)	1 (5)	12 (63)	
**n (%)**					
***BRAF wild type***	112 (97)	9 (50)	18 (95)	7 (37)	
**n (%)**					

### Clinical correlations

**Fig 1:**
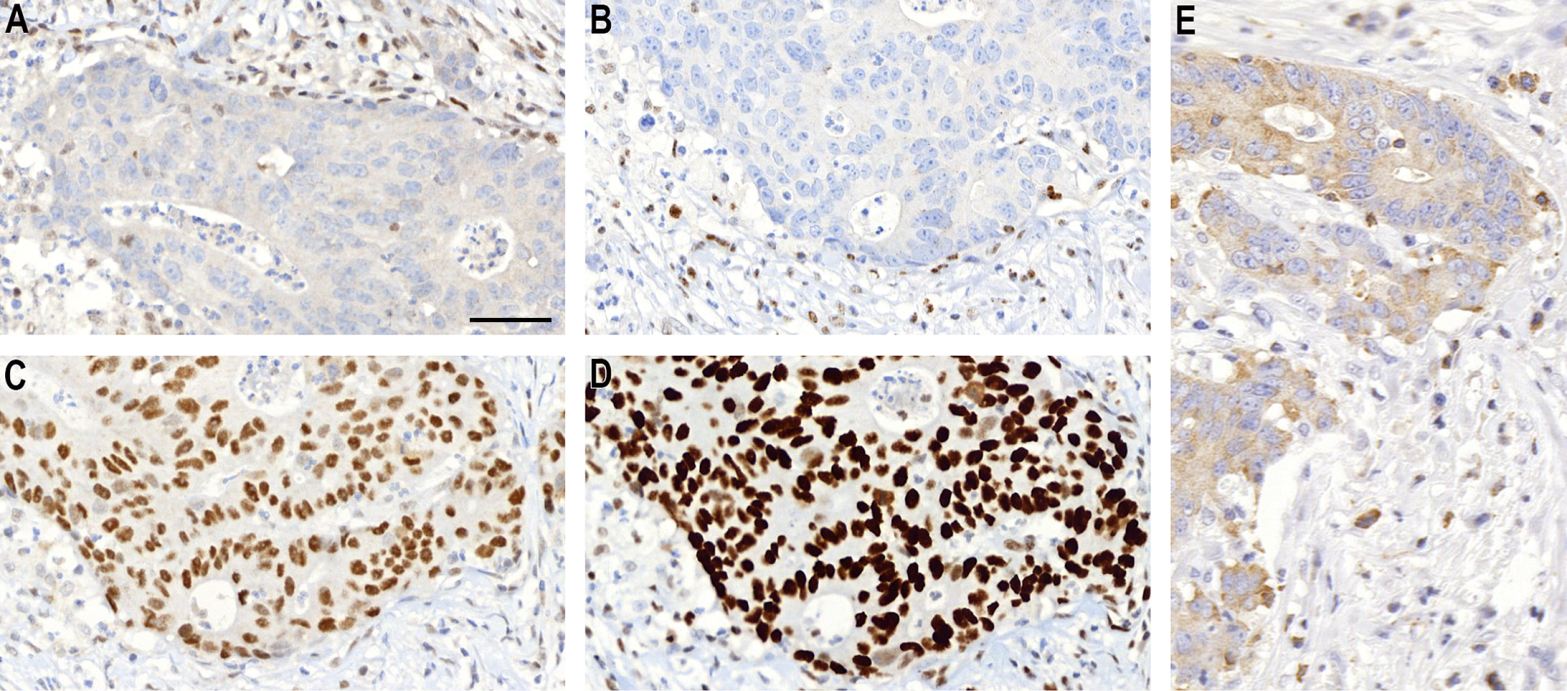
An example of a tumor with *MSI*-high features and weak ezrin expression. MLH1 (A) and PMS2 (B) are negative in nuclei of cancer cells, while MSH2 (C) and MSH6 (D) show normal nuclear staining. Tumor cells show weak immunostaining for ezrin (E). This patient had a favorable prognosis.

**Fig 2:**
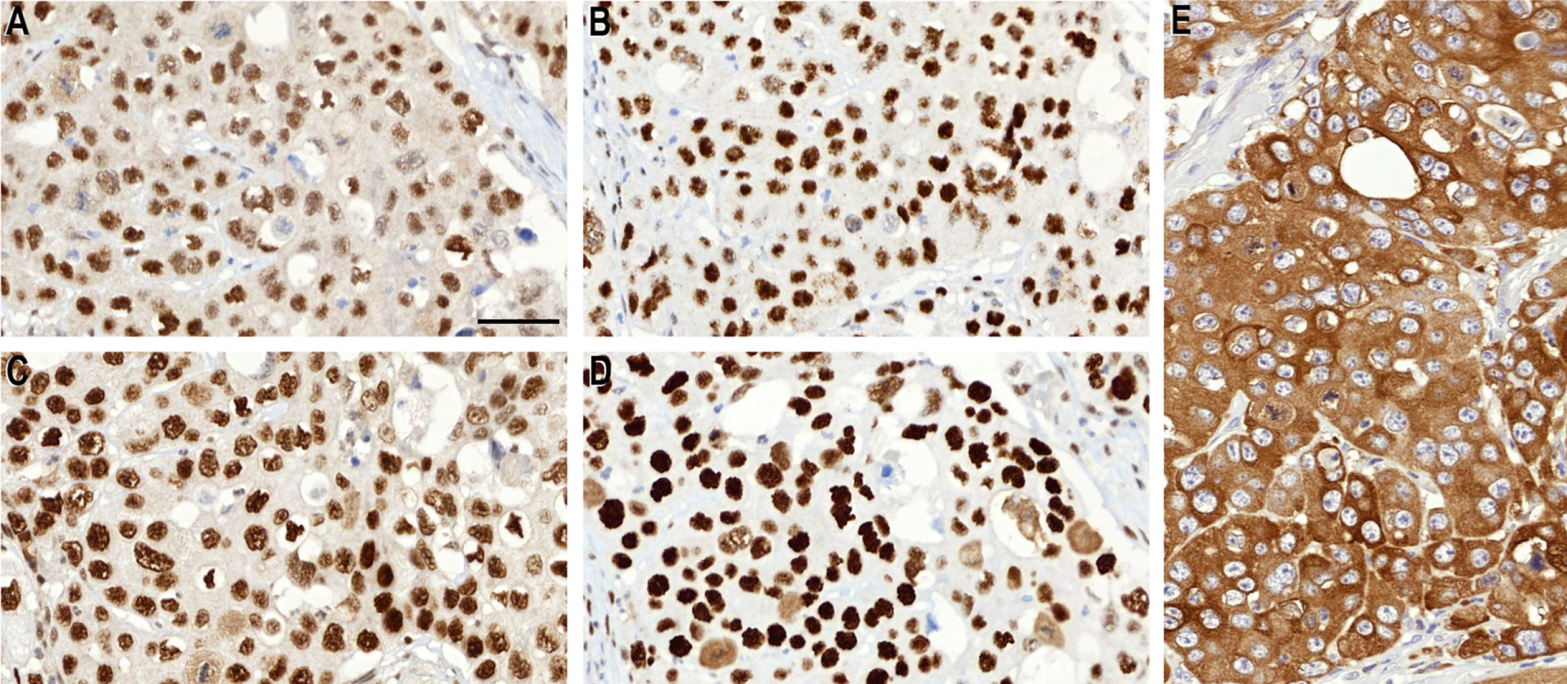
An example of a tumor with MSS features and strong ezrin expression. MLH1 (A), PMS2 (B), MSH2 (C) and MSH6 (D) show normal nuclear staining in colorectal cancer cells. Tumor cells show strong immunostaining for ezrin (E). This patient had an unfavorable outcome.

**Fig 3:**
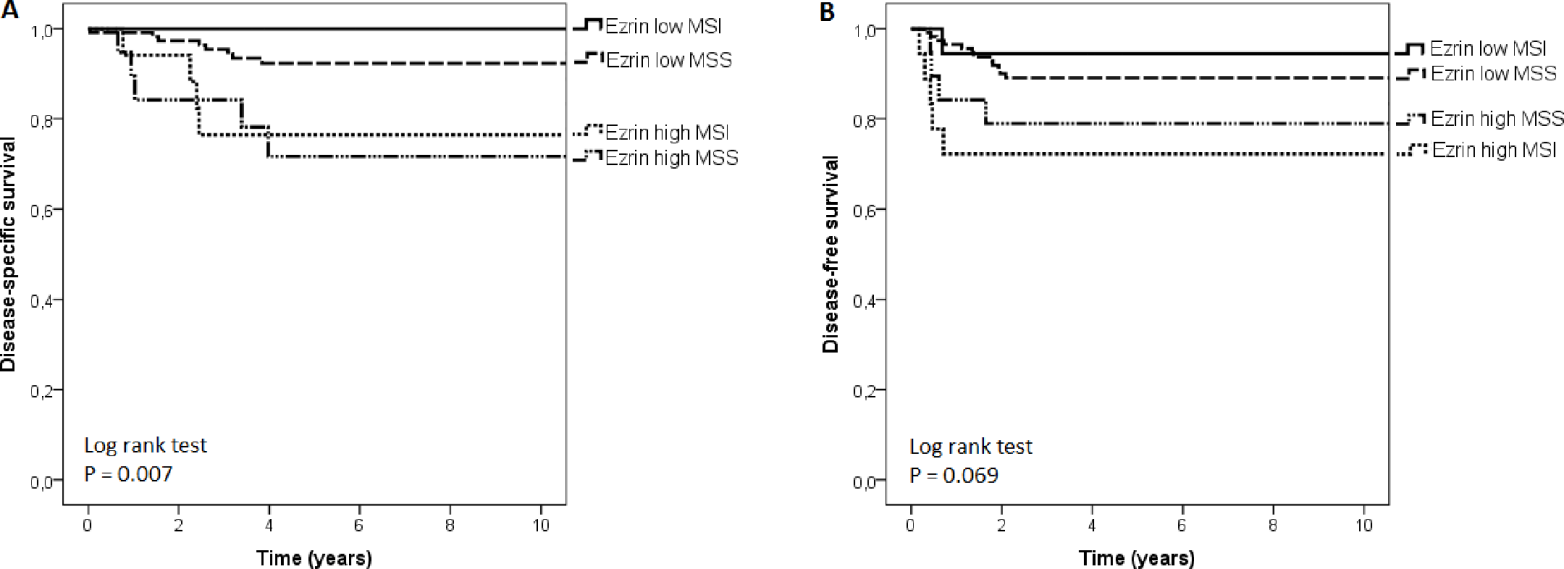
Kaplan-Meyer survival analysis of stage II colorectal cancers based on *MSI* status and ezrin expression. Disease-specific survival (A) and disease-free survival (B).

The clinical correlations of the *MSI* status and ezrin staining are shown in Tables 2 and 3. Altogether, high ezrin staining correlated with inverse DSS (Fisher’s exact test, p = 0.038). On the other, *MSI* status as a single variable did not correlate with survival. In categorical analysis of 5-year disease-specific survival time, 11 out of 18 (61%) patients with *MSI* / low ezrin were alive compared to only 4 out of 18 (21%) patients with *MSS* / high ezrin (Fisher’s exact test, p = 0.040). In univariate analysis, patients whose tumors were *MSI* / low ezrin tended to have the best OS probability, and those with *MSI* / high ezrin the worst, but the difference was not statistically significant (log-rank test, p = 0.235). Patients with *MSI* / low ezrin tumors had the longest DSS and those with *MSS* / high ezrin tumors had the shortest (log-rank test, p = 0.007). An example of staining patterns of patients belonging to groups of best and worst DSS are presented in Figs 1 and 2, respectively. Patients with *MSI* / low ezrin had the longest DFS and those with *MSI* / high ezrin had the shortest, but the difference was no statistically significant (log-rank test, p = 0.069). The survival curves are presented in Fig 3 and the results of univariate survival analysis in S1 Table.

A summary of the multivariate analyses results is presented in Table 5. This table shows T4bN0 tumors to be associated with inferior OS (Cox model, HR 2.86, 95% CI [1.06–7.74], p = 0.038) and DFS (Cox model, HR 8.05, 95% CI [2.31–28.01], p = 0.001). Likewise, perforation was linked to inferior OS (Cox model, HR 3.8, 95% CI [1.57–9.17], p = 0.003), DSS (Cox model, HR 5.44, 95% CI [95% CI 1.3–22.75], p = 0.02), as well as DFS (Cox model, HR 4.87 95% CI [1.38–17.23]; p = 0.014). Moreover, the presence of *BRAF* mutation was associated to shortened OS (Cox model, HR 3.29, 95%CI [1.14–9.54], p = 0.028). High ezrin expression together with *MSS* were linked to shorter DSS (Cox model, HR 5.68, 95%CI [1.53–21.12], p = 0.01).

**Table 5:** Summary of the results in multivariate analysis.

Variable (n)	Overall survival			Disease-specific survival			Disease-free survival		
	HR	95% CI	*p value*	HR	95% CI	*p value*	HR	95% CI	*p value*
**Stage T3N0 (143)**	Reference category			Reference category			Reference category		
**Stage T4aN0 (17)**	1.76	0.64–4.83	0.275	3.40	0.72–15.98	0.121	3.04	0.82–11.33	0.097
**Stage T4bN0 (13)**	2.86	1.06–7.74	**0.038**	4.58	0.89–23.62	0.069	8.05	2.31–28.01	**0.001**
**Grade 1 (19)**	Reference category			Reference category			Reference category		
**Grade 2 (114)**	0.50	0.20–1.29	0.153	0.93	0.13–6.56	0.946	0.82	0.13–5.21	0.838
**Grade 3 (40)**	0.53	0.18–1.53	0.241	0.68	0.08–6.12	0.732	1.27	0.18–8.87	0.809
**Right colon (89)**	1.35	0.69–2.65	0.378	1.27	0.37–4.36	0.702	1.04	0.39–2.83	0.933
**Vascular invasion (19)**	1.57	0.78–3.18	0.210	3.36	0.98–11.57	0.055	3.62	1.26–10.37	**0.017**
**Perforation (10)**	3.80	1.57–9.17	**0.003**	5.44	1.3–22.75	**0.002**	4.87	1.38–17.23	**0.014**
**Preop. obstruction (19)**	0.71	0.27–1.85	0.479	1.32	0.31–5.65	0.71	1.53	0.45–5.21	0.499
***BRAF* mutation (12)**	3.29	1.14–9.54	**0.028**	1.41	0.20–9.90	0.728	1.00	0.20–5.07	0.997
**Ezrin low *MSS (117)***	Reference category			Reference category			Reference category		
**Ezrin low *MSI (18)***	0.34	0.10–1.15	0.083	0.00	0.00-.000	0.986	0.78	0.09–6.66	0.824
**Ezrin high *MSS (19)***	0.98	0.37–2.64	0.975	5.68	1.53–21.12	**0.01**	2.76	0.76–1.01	0.124
**Ezrin high *MSI(19)***	0.76	0.26–2.21	0.619	3.19	0.61–16.74	0.17	3.01	0.78–11.66	0.110

## Discussion

Stage II CRC patients possess a treatment challenge, because current diagnostic methods do not enable their accurate risk stratification. The purpose of this study was to test, whether analysis of ezrin, a promising prognostic marker, together with microsatellite instability and *BRAF* mutation status could be used for prognostication. Indeed, our results show ezrin as an independent prognostic marker for disease-specific survival in stage II CRC, and indicate this correlation to be further strengthened by concomitant microsatellite instability testing.

Previous studies by others and us have indicated an association between ezrin expression and CRC outcome. The earlier studies have been carried out with mixed cohorts, including various disease stages, which can lead to inaccurate conclusions. However, we are not aware of any studies that would have specifically focused on ezrin expression in stage II CRC. The current results indicate that tumors with high ezrin expression possess adverse biological features already at a stage, when the cancer has not yet disseminated. Importantly, as demonstrated by this study, these features are not associated with tumor location, histological grade, vascular invasion or other outcome-related clinicopathological features. Our results do not clarify the mechanism, by which ezrin may be linked with oncogenic properties. One explanation is that ezrin expression provides an advantage for the disseminating cells early on during metastatic seeding. Indeed, some previous studies have indicated a role for ezrin in this process [32,33]. Interestingly, ezrin turned out to be a stronger DSS predictor than any of the clinipathological factors, apart from tumor perforation.

Ezrin expression is linked to the activity of several oncogenic signaling cascades. Ezrin can act both as a regulator and/or a down-stream target in several signaling pathways, including *Src*, *Akt*-*PI3K* and *PKA*, and these associations have been suggested to be of importance in ezrin’s oncogenic properties [34,35,36]. Here, we found that ezrin expression correlated with *BRAF* mutation status; high ezrin immunoreactivity being significantly more common in *BRAF V60*0E than *BRAF wild-type* tumors. This is a novel finding, there are no previous reports that would have linked ezrin with *BRAF*. Even if the specific mechanism of the connection between these two genes is unknown, the association of both high ezrin expression and BRAF mutation with the activity of several oncogenic signaling pathways might partly explain this interesting finding.

In this study, *MSI* status alone did not correlate with survival, although the superior prognosis of patients with *MSI* CRC over *MSS* tumors has been demonstrated earlier in many studies [37,38,13,39,21]. However, the combination of ezrin expression with *MSI* status stratified the patients to prognostic groups, in which patients with *MSS* and high ezrin expression had the shortest DSS and patients with *MSI* and low ezrin expression had the best DSS (log-rank test, p = 0.007). This correlation is of interest as high ezrin expression was significantly more infrequent in *MSS* tumors than *MSI* tumors. Why the prognostic role of ezrin is especially pronounced in *MSS* tumors awaits further studies.

With this university hospital area based cohort we could confirm earlier findings related to microsatellite instability and *BRAF* mutation status. Mucinous histology and poor differentiation grade were associated with *MSI*, which is in accordance with *MSI* high phenotype [40]. In the current study, about a fifth of the tumors were *MSI* high, and *MSI* high tumors were significantly more commonly right-sided, as reported previously for stage II tumors [37,13]. Sidedness in itself, however does not justify patient selection for possible adjuvant therapy in stage II CRC [41].

In the current study, 84% of *BRAF mutated* tumors were *MSI*, whereas most *BRAF wild ty*pe tumors were *MSS*. *BRAF* mutation was also significantly linked to overall survival, the HR for mortality being 3.29 (95%CI [1.14–9.54], p = 0.028). Similar results have also been reported in previous studies, showing *BRAF* mutation to associate with increased mortality due to CRC [21,42]. There is evidence that *MSI* phenotype may compensate the poor prognostic effect of *BRAF* mutation [43], but this issue remains controversial [44]. *BRAF* mutation is also reported to rule out Lynch syndrome [17], which may be of help to the clinicians in counseling the patients and their families.

At the time-point the patients were treated, *MSI*-status was not routinely tested among stage II patients. Altogether, 37% of the patients had received adjuvant chemotherapy, according to possible high-risk factors including preoperative obstruction or perforation, vascular invasion, poor differentiation grade and T4-stage and depending on their overall health, general health and patient preference. Patients with *MSI* tumors are reported not to gain benefit from fluorouracil-based adjuvant chemotherapy [13,45]. This concerns especially stage II colorectal cancer patients, while there are conflicting results concerning stage III patients [46].

In conclusion, our study found a correlation between ezrin expression and DSS in stage II CRC, and this correlation was further strengthened by microsatellite instability analysis. Of the different tumor categories, DSS was longest in patients presenting with MSI / low ezrin tumors and shortest in *MSS* / high ezrin tumors. These results imply that ezrin staining can provide important prognostic information for estimating stage II patients’ individual risk of disease recurrence and progression.

## Supporting information

S1 Table Univariate analysis survival data according to clinicopathological parameters and biomarkers. (DOCX)
