# Supplementary material for: Ezrin expression combined with MSI status in prognostication of stage II colorectal cancer
Source: PLoS One. 2017 Sep 27;12(9):e0185436. doi: 10.1371/journal.pone.0185436 (PMC5617236; doi:10.1371/journal.pone.0185436)
Supplement: S1 Table — (DOCX) [file pone.0185436.s001.docx]

**S1 Table**. Univariate analysis survival data according to clinicopathological parameters and biomarkers.

| **Variable** | **Patients** | **Overall survival** | **Disease-specific survival** | **Disease-free survival** |
| --- | --- | --- | --- | --- |
|  | **(n)** | **HR (95% CI) *p* value** | **HR (95% CI) *p* value** | **HR (95% CI) *p* value** |
| StageT3N0 | 143 | 1 Reference category | 1 Reference category | 1 Reference category |
|  |  |  |  |  |
| Stage T4aN0 | 17 | 1.2 (0.47-3.04) 0.703 | 2.35 (0.65-8.41) 0.19 | 3.02 (0.97-9.35) 0.056 |
|  |  |  |  |  |
| Stage T4bN0 | 13 | 2.51 (1.2-5.6) **0.025** | 3.96 (1.1-14.24) **0.035** | 7.88 (2.94-21.11) **<0.001** |
|  |  |  |  |  |
| Grade 1 | 19 | 1 Reference category | 1 Reference category | 1 Reference category |
|  |  |  |  |  |
| Grade 2 | 114 | 078 (0.33-1.86) 0.573 | 0.87 (0.19-3.94) 0.859 | 1.15 (0.26-5.1) 0.853 |
|  |  |  |  |  |
| Grade 3 | 40 | 0.93 (0.35-2.43) 0.878 | 0.99 (0.18-5.4) 0.989 | 1.1 (0.4-9.21) 0.419 |
|  |  |  |  |  |
| Right colon | 89 | 1.31 (0.76-2.26) 0.326 | 1.05 (0.4-2.71) 0.926 | 0.92 (0.4-2.13) 0.852 |
|  |  |  |  |  |
| Vascular invasion | 32 | 1.29 (0.68-2.46) 0.437 | 1.76 (0.62-4.99) 0.29 | 2.00 (0.82-4.92) 0.129 |
|  |  |  |  |  |
| Perforation | 15 | 4.08 (1.96-8.52) **<0.001** | 6.44 (2.25-18.41) **0.001** | 7.24 (2.93-17.9) **<0.001** |
|  |  |  |  |  |
| Preoperative | 26 | 0.76 (0.32-1.78) 0.526 | 1.75 (0.57-5.36) 0.33 | 2.23 (0.87-5.7) 0.094 |
| obstruction |  |  |  |  |
| Examined lymph | 35 | 0.64 (0.31-1.31) 0.636 | 1.13 (0.37-3.46) 0.835 | 0.84 (0.28-2.47) 0.744 |
| nodes < 12 |  |  |  |  |
| Ezrin high | 38 | 1.69 (0.93-3.06) 0.087 | 4.47 (1.72-11.59) **0.002** | 2.79 (1.19-6.52) **0.018** |
|  |  |  |  |  |
| MSI high | 37 | 1.2 (0.65-2.21) 0.56 | 1.14 (0.37-3.5) 0.818 | 1.46 (0.57-3.74) 0.427 |
|  |  |  |  |  |
| BRAF mutation | 25 | 1.9 (1.01-3.55) **0.045** | 1.46 (0.42-5.12) 0.556 | 1.04 (0.31-3.54) 0.948 |
|  |  |  |  |  |
| Ezrin low MSS | 117 | 1 Reference category | 1 Reference category | 1 Reference category |
|  |  |  |  |  |
| Ezrin low MSI | 18 | 0.75 (0.29-1.91) 0.543 | 0 (0-0) 0.983 | 0.51 (0.07-3.92) 0.518 |
|  |  |  |  |  |
| Ezrin high MSS | 19 | 1.29 (0.54-3.07) 0.571 | 4 (1.31-12.23) **0.015** | 2.14 (0.69-6.63) 0.188 |
|  |  |  |  |  |
| Ezrin high MSI | 19 | 1,95 (0.93-4.06) 0.076 | 3.6 (1.08-11.96) **0.037** | 3.13 (1.1-8.9) **0.032** |
|  |  |  |  |  |
| Ezrin low BRAF wt | 123 | 1 Reference category | 1 Reference category | 1 Reference category |
|  |  |  |  |  |
| Ezrin low BRAFmut | 12 | 1.61 (0.67-3.85) 0.287 | 0 (0-0) 0.98 | 0 (0-0) 0.982 |
|  |  |  |  |  |
| Ezrin high BRAF wt | 25 | 1.43 (0.66-3.11) 0.363 | 3.99 (1.38-11.5) **0.01** | 2.50 (0.94-6.53) 0.066 |
|  |  |  |  |  |
| Ezrin high BRAF mut | 13 | 2.5 (1.10-5.61) **0.029** | 4.25 (1.13-16.05) **0.033** | 2.65 (0.76-9.31) 0.128 |
|  |  |  |  |  |
